# Determination of Risk Factors Associated with Foot and Mouth Disease Outbreaks in Dairy Farms in Chiang Mai Province, Northern Thailand

**DOI:** 10.3390/ani10030512

**Published:** 2020-03-19

**Authors:** Chalutwan Sansamur, Orapun Arjkumpa, Arisara Charoenpanyanet, Veerasak Punyapornwithaya

**Affiliations:** 1 Department of Veterinary Biosciences and Veterinary Public Health, Faculty of Veterinary Medicine, Chiang Mai University, Chiang Mai 50100, Thailand; chalutwan_s@cmu.ac.th (C.S.); orapun_arj@cmu.ac.th (O.A.); 2 Department of Geography, Faculty of Social Sciences, Chiang Mai University, Chiang Mai 50200, Thailand; arisara.cmu@gmail.com; 3 Veterinary Public Health and Food Safety Centre for Asia Pacific (VPHCAP), Faculty of Veterinary Medicine, Chiang Mai University, Chiang Mai 50100, Thailand

**Keywords:** risk factors, dairy farm, foot and mouth disease, biosecurity, northern Thailand

## Abstract

**Simple Summary:**

One hundred and 40 dairy farms that experienced foot and mouth disease (FMD) outbreaks and 307 farms without FMD outbreaks were investigated in this research study. Relevant farm owners were interviewed in order to determine the farm-level risk factors associated with the FMD outbreaks. We established that the risk factors for FMD outbreaks were (1) purchasing a new cow without following quarantine protocol, (2) FMD vaccination administration by non-official livestock personnel, (3) farms located within a 5 km radius of cattle abattoirs, (4) farms located near shared cattle grazing areas in a 10 km radius and (5) no history of FMD outbreaks in the previous year. Most of the risk factors were related to indirect transmissions of FMD and biosecurity practices, thus we have advised dairy farmers to strengthen management practices associated with FMD prevention protocols.

**Abstract:**

Foot and mouth disease (FMD) is considered a highly contagious transboundary disease of cloven-hoofed animals. FMD has become endemic to northern Thailand over the past decade. In 2016, FMD outbreaks were recorded in three districts in Chiang Mai Province. The objective of this study was to determine the farm-level risk factors associated with FMD outbreaks. This study was conducted via a face-to-face interview questionnaire survey at 140 FMD outbreak farms and 307 control farms. Univariable and multivariable logistic regression analyses were used to determine the association between potential risk factors and FMD outbreaks. The final logistic regression model identified factors associated with FMD outbreaks including the purchasing of a new cow without following quarantine protocol (odds ratio = 2.41, 95%CI = 1.45, 4.05), farms located near shared cattle grazing areas in a 10 km radius (OR = 1.83, 95%CI =1.11, 3.02), FMD vaccination administration by non-official livestock personnel (OR = 2.52, 95%CI = 1.39, 4.58), farms located in a 5 km radius of cattle abattoirs (OR = 1.83, 95%CI = 0.99, 3.40) and no history of FMD outbreaks over the previous 12 months in districts where farms were located (OR = 0.44, 95%CI = 0.22, 0.86). The risk factors identified in this study were related to farm biosecurity, FMD vaccination administration and distance from the farms to risk areas. Therefore, it was important to strengthen on-farm biosecurity and to improve farm management practices in order to reduce incidences of FMD at the farm level. Education or training programs for dairy farmers that would enhance knowledge and practices in relation to the assessed topics are needed.

## 1. Introduction

Foot and mouth disease (FMD) is considered to be one of the world’s most important livestock diseases, and is a highly contagious transboundary disease of cloven-hoofed animals, including livestock and wildlife [1]. It has been classified as a multiple species disease. It is a disease that is rapidly and extensively spreading between countries and can have a severe economic and social impact according to the World Organization for Animal Health (OIE) [2]. Due to losses in milk production, fertility problems and potential changes in herd structure as a result of poor proportions of breeding animals within herds, this disease can cause major losses in dairy production [3]. FMD is caused by a virus in the Picornaviridae family and induces vesicles on the feet, mammary glands and in the infected animal’s oral cavity [4,5]. The FMD virus (FMDV) can be transmitted via different routes (i.e., direct or indirect contact between infected and susceptible animals, their secretions, contaminated animal products, etc.) [6].

FMD is endemic worldwide and has been predominantly detected in a variety of livestock species in Southeast Asia [7]. Over the past decade (2007–2017), there have been 4961 recorded FMD outbreaks in Cambodia, Lao People’s Democratic Republic, Malaysia, Myanmar, Vietnam and Thailand. These outbreaks have involved a number of debilitating incursions. Notably, alerts of FMD outbreaks between 2007 and 2016 in Thailand, one of Southeast Asia’s member countries, increased by approximately five-fold from 42 to 240 reports [8]. FMD control measures currently applied in Thailand include the control of the movement of animal and animal products during an outbreak, the administration of ring vaccinations, animal quarantine policies, environmental sanitary controls, outbreak investigations, serological surveillance and proper disposal of carcasses and contaminated materials and campaigns directed at improving public awareness [9]. Full knowledge of the relevant risk factors is a key component of implementing an effective control strategy. Some research studies in Southeast Asia have provided information on FMD risk factors for cattle. For instance, questionnaire-based case–control studies have been widely used to gather information on the risk factors associated with FMD spread in certain countries. The established risk factors include livestock transportation methods [10], the sharing of grazing areas [11], the proximity of the farm to the main road [12] and the mixing of animals within public pastures [13].

As northern Thailand shares borders with Myanmar and Lao PDR, it is an important area for livestock production. This is particularly true with regard to the dairy and beef cattle industries, which are especially susceptible to FMD [14]. Traditionally, dairy cattle are tied and kept in stalls in the barn, while the beef cattle industry employs free-range grazing practices [15]. Previous studies have suggested that the risk factors for FMD in ruminants in northern Thailand include the distance to the nearest abattoir, animal and vehicle movements, proportions of vaccinated animals and shared animal grazing and watering locations [16,17]. Disease investigation data on FMD outbreaks during the years 2008–2015 were predominantly related to animal and equipment movement on farms, artificial insemination practices, the use of public pastures, the use of canals or ponds as main water sources, contaminated animal feed and the contamination that often occurs in abattoirs [18]. Dairy production is the major livestock activity in this region, primarily in Chiang Mai Province. Dairy and household populations in Chiang Mai were recorded at 30,667 heads of cattle and 1070 households. The average milk yield from all farms per month was 6,284,970 kg, which is approximately 59% of the milk yield production for all of northern Thailand [19]. Notably, this province experienced a huge FMD outbreak in 2016 (number of outbreak = 26 times). This incident was remarkably catastrophic when compared with 2017–2019 (number of outbreaks = eight times) [20]. According to investigative data obtained from the Department of Livestock Development (DLD), most of the farms in the area that experienced an FMD outbreak in 2016 were dairy farms. Formal disease outbreak investigations were performed by DLD officers; however, there have been no scientific reports or research publications produced on these outbreaks. Moreover, any analysis of the relevant risk factors based on inferential statistics has been very limited. It is very important to work from an updated body of knowledge and to learn from past experiences in order to fully understand the outbreak circumstances and the potential risk factors. Therefore, the objective of this study was to quantify the risk factors associated with FMD outbreaks on dairy farms that occur at the farm level. This research has the benefit of updating the current prevention and control measures in place at the farm level with regard to endemic FMD outbreaks in northern Thailand.

## 2. Materials and Methods

### 2.1. Study Area

Chiang Mai Province is situated in northern Thailand at a latitude of 18°47′25.37″ N and longitude of 98°59′4.85″ E. In total, there were 26 outbreaks occurred in 10 districts in the period of July to November 2016. There were 667 dairy cattle were diagnosed as FMD infected animals. The majority of the outbreak was caused by FMD virus serotype O [20]. The local DLD staffs implemented FMD control measures including (1) quarantine of the suspected animals, (2) submission of a sample of tissue lesions to the laboratory, (3) restriction of animal and animal product movement in the outbreak area for 30 days, (4) conduct a ring vaccination, (5) cleaning and spraying vehicles at control point, (6) disposal of carcasses and contaminated materials and (7) increasing public awareness [9] In this study, we selected 3 districts including Mae On, San Kamphaeng and San Sai districts because these 3 districts were located in close proximity to each other and contained a large number of dairy farms. Officially, there were 5 episodes of FMD outbreaks in 3 districts in the study area based on the DLD investigations conducted during the incursion periods.

### 2.2. Data Collection

#### 2.2.1. Official FMD Outbreak Data and Case Definition

FMD outbreak investigative data recorded in the three study districts were retrieved from the Chiang Mai Provincial Livestock Office. The FMD outbreak farms were defined as dairy farms in which at least one animal was recorded with typical clinical signs of FMD, including vesicles on the feet, mammary glands and around the oral cavity in 2016 [21] by district livestock officers, or tissue sample from animals with typical FMD signs was confirmed as being FMDV positive by applying enzyme-linked immunosorbent assay techniques at the Veterinary Research and Development Center Upper Northern Region, Lampang Province, Thailand [20].

#### 2.2.2. Field Data Collection and Variables

We collected data with the use of a questionnaire survey distributed among all dairy farms registered to dairy cooperatives and/or Chiang Mai Provincial Livestock Office in the three study districts. After all outbreaks, a questionnaire survey was done for all farms in the study areas. All farmers participated in the survey. FMD outbreak farms (*n* = 140) and FMD non-outbreak farms (*n* = 307) were visited by one veterinarian and an authorized livestock officer. The survey covered events from February to June 2017. The questionnaire was designed to determine the influence of the plausible risk factors on the likelihood of a farm being infected and was assessed and pre-tested among 45 farmers in order to obtain a primary-standardized questionnaire. The questionnaire was composed of 5 parts including: (1) general information on farmers, (2) farm husbandry and environment, (3) animal and vehicle movement before the outbreak, (4) historical FMD infection and vaccination status and (5) biosecurity practices during FMD outbreaks. In addition, geographical coordinates (X–Y coordinates) were recorded when visiting the farms. 

#### 2.2.3. Disease Mapping

The spatial data including administrative details (district level), data on infrastructure (roadways) and information on watering areas were received from the Geo-Informatics and Space Technology Centre (Northern Region), Faculty of Social Sciences, Chiang Mai University. Variables in the geographic class were thought to influence the spatial distribution of FMD. The risk factors related to the distance between dairy farms and beef farms, the road, cattle and pig abattoirs, milk collecting centers and grazing locations were assessed to create a vector map and then employed to identify the relevant factors for analysis. The cut-off point values of the specific distances must also be justified as follows: distance within a 500 m radius between a farm and neighboring dairy farms, beef farms and roads based on previous studies [12,22]; a distance within a radius of 5 km between farm and cattle, pig abattoirs and milk collecting centers and a distance within a radius of 10 km between farm and grazing locations based on DLD authority for FMD control measures [9] (Table S1). Disease mapping was generated using QGIS version 2.18.28 (Open Source Geospatial Foundation Project, Zurich, Switzerland) [23]. A map was produced indicating the 2016 FMD outbreak farms and the FMD non-outbreak farms in each district.

### 2.3. Statistical Analysis

The association between FMD outbreak status and the relevant risk factors was analyzed with univariable and multivariable logistic regression by R statistical software version 3.6.2 (R Core Team, Vienna, Austria) [24] using the package “stats” [24] and “rms” [25]. Initially, for univariable analysis, the chi-square test was performed to determine the relationship between FMD status and the relevant risk factors. In addition, an odds ratio (OR) with a 95% confidence interval (CI) was used to calculate each categorical risk factor. Factors with a significance level of *p* ≤ 0.2 from the univariable analysis were selected for multivariable logistic regression analysis. All selected risk factors were checked for multicollinearity using the chi-square test for categorical variables. In the event of multicollinearity (*p* < 0.05), the factor with higher biological acceptability was retained for multivariable logistic regression analysis. The form of the multivariable logistic regression model was achieved using the following equation:

(1) $$In\left(\frac{P_{i}}{1-P_{i}}\right)=\beta_{0}+\beta_{1}X_{1}+\cdots +\beta_{k}X_{k}$$

where ***P_i_*** is the probability of an FMD outbreak on the farm *i* (*i* = 1,.., 447), ***X_k_*** is a set of risk factors (***X_k_*** = 1,.., k) and $\beta_{k}$ is the estimated coefficient for the risk factors $(\beta_{k}\;$= 1,..,k).

For the multivariable analysis, the variables that were determined to be significant at *p* < 0.2 in the univariable analysis were included for model selection. The options for model selection were set using a backward direction based on the Akaike Information Criterion (AIC). The model with the lowest AIC was justified as the final model. The overall model fitting of the final model was tested using the Hosmer–Lemeshow test [26]. The ability of the model to discriminate between an FMD outbreak farm and a non-FMD outbreak farm was tested using the Receiver Operating Characteristic (ROC) method. The general rules of the area under the ROC curve (AUC) were: AUC = 0.5 (no discrimination), 0.5  <  AUC  <  0.6 (poor discrimination), 0.6  ≤  AUC  <  0.7 (fair discrimination), 0.7 ≤ AUC < 0.8 (acceptable discrimination), 0.8 ≤ AUC < 0.9 (excellent discrimination) and AUC ≥ 0.9 (outstanding discrimination) [27].

### 2.4. Ethical Statements

The authors confirm that the ethical policies of the journal, as noted on the journal’s author guidelines page, have been adhered to and the appropriate ethical review committee approval has been received. Chiang Mai University Research Ethics Committee has reviewed and approved of the research proposal based on international guidelines for human research protection (COA No. 008/60). Participants of the questionnaire survey were dairy farmers who voluntarily gave informed consent to participate in the interviews. There were no animal samples collected in this study.

## 3. Results

### 3.1. Descriptive Statistics

**Figure 1:**
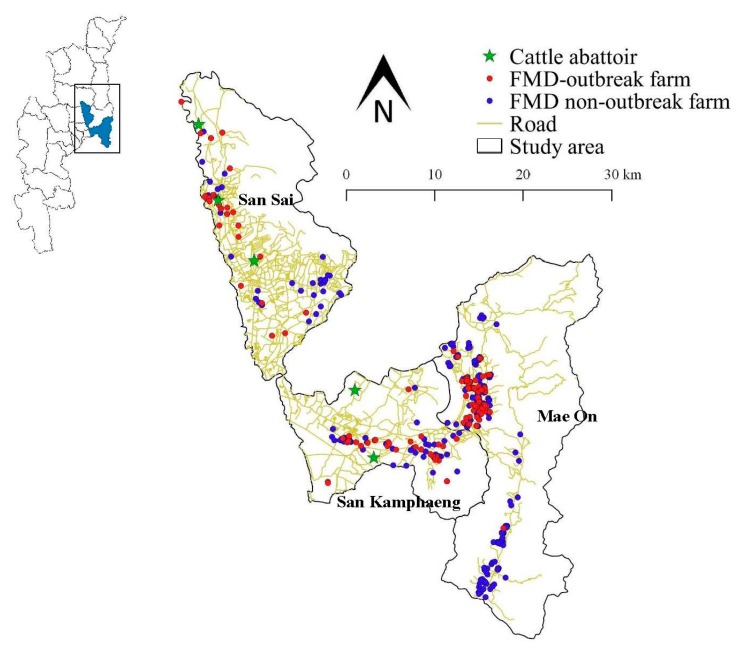
Location of foot and mouth disease (FMD) outbreak farms (red points), FMD non-outbreak farms (blue points), location of cattle abattoirs (green points) and roadways (gold lines) in 3 districts of Chiang Mai Province, northern Thailand.

A total of 447 farms were included in this study at the following distribution values: 62.6% in Mae On (*n* = 280), 22.8% in San Kamphaeng (*n* = 102) and 14.5% in San Sai districts (*n* = 65), with an average ownership of 33 dairy cattle for each farm (SD = 18.64). The number of FMD outbreak farms in Mae On, San Kamphaeng and San Sai districts were 74, 35 and 31, respectively. Farm locations were mapped and are presented in Figure 1.

Most respondents were male (82.3%). Approximately half of the participants were older than 40 years old. Around two-thirds of the respondents were educated at the primary school level. Sixty-three percent of dairy farmers had more than 10 years’ experience in their careers.

All dairy farms utilized the tie-stall system. Most of the farms (70.5%) were located within a 5 km radius of the milk collecting centers. All dairy farmers transported bulk milk in tanks to their dairy cooperatives in the mornings and evenings of every day via their own vehicles (*n* = 345) or by publicly shared milk collecting vehicles (*n* = 102). All dairy farms used artificial insemination (AI) services for the breeding of their livestock. For all farms, FMD vaccinations were applied three times per year (1) from December to January, (2) April to May and (3) August to September. However, some dairy farms that had administered FMD vaccinations to livestock did not apply the vaccine to all cattle on the farm (*n* = 29). Some FMD outbreak farms were located near cattle abattoirs (*n* = 9), roadways (*n* = 67) and near both cattle abattoirs and roadways (*n* = 36) (see Table S1). Information on general management practices, animal and vehicle movements, vaccinations of animals and farm biosecurity data obtained from the questionnaire survey are shown in Table 1.

### 3.2. Risk Factors

**Figure 2:**
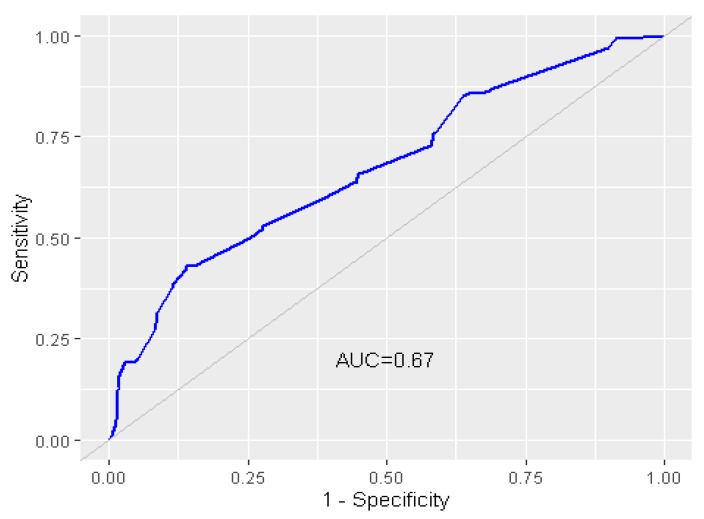
Predicted probabilities of final model based on the Receiver Operating Characteristic (ROC) method (the area under the ROC curve (AUC) = 0.67).

There were seven significant factors acquired from the univariable analysis with *p* < 0.05 (Table 2). However, with the criteria of *p* < 0.2, 14 factors were included in the model selection process for multivariable analysis (Table S2). With regard to multicollinearity, we found that three pairs of variables produced a multicollinearity result that involved collinearity between (1) purchase of a new cow without following quarantine protocol and the entrance of vehicles carrying stock and/or cows out of the farm, (2) the use of tap water on farms and farms located near shared cattle grazing areas in a 10 km radius and (3) farms located in a 5 km radius of cattle abattoirs and farm located near roadways (see Table S3). The variables with higher biological plausibility were retained for multivariate analysis including purchasing of a new cow without following quarantine protocol, farms located near shared cattle grazing areas in a 10 km radius and farms located in a 5 km radius of cattle abattoirs. Therefore, these variables were considered under the multivariable analysis procedure. Based on the AIC criteria, the final model included six factors that are shown in Table 3. The final model accounting for all risk factors achieved a good fit (Hosmer–Lemeshow test, *p* = 0.08). The accuracy of the final model was assessed by ROC and showed fair discrimination (AUC = 0.67, Figure 2).

It is important to note that we included dairy farms from 3 districts in this study. The occurrence of FMD outbreaks was often different; therefore, the effects of each district on the FMD outbreak were tested in the statistical model. This factor was determined to be a significant factor based on univariable analysis; therefore, we included this factor for the final model selection. Two models including a model with district factor and one without district factor were generated and compared. The model without district factor did not fit to the data (Hosmer–Lemeshow test, *p* = 0.01). In addition, the model with district factor (AIC = 524.89) was found to be better than the model without this factor (AIC = 529.14). Therefore, in terms of interpretation, the OR for each factor in our final model accounted for the effects of the farming area.

## 4. Discussion

This study identified the farm-level risk factors associated with the FMD outbreaks in Chiang Mai Province, northern Thailand. It was found that the significant factors were related to farm biosecurity, FMD vaccination administration and the distance from the farm to the risk area.

It is well known that animal and vehicle movements are risk factors that are associated with endemic transmissions [11,28]. As evidenced by various previous studies, the movement of cattle was significantly associated with FMD outbreaks [10,29,30]. Similarly, we found that purchasing a new cow on a farm without observing a quarantine protocol increased the risk of an FMD outbreak by 2.41 times. In the study area, dairy cattle were commonly transported to farms by dealers without any pre-movement testing and dairy cattle were not usually quarantined at the destination farm; hence the FMD virus could be transmitted from newly purchased dairy cattle to susceptible cattle already in existence on the farm.

Our results indicated that farms located near shared cattle grazing areas were associated with FMD outbreaks. Despite previous studies conducted in other countries showed that the sharing of grazing or water sources is a risk factor for FMD outbreaks [16,30,31], neither was associated with the occurrence of FMD in this study. It was unclear why dairy farms that were located near cattle grazing areas revealed a higher degree of risk because the direct contact between beef and dairy cows was very limited. In addition, all dairy farms in the study area employed the tie-stall system and all of these farms followed the farm standard regulations regarding Good Agriculture Practices (GAP) for livestock [32], in which it is stated that all dairy farms must utilize a fencing structure. However, it may be postulated that the airborne spreading of the virus could have occurred as has been described in the previous study [33]. Moreover, we found that farms located near a cattle abattoir had a higher risk of FMD occurrences. This finding was also in accordance with those of previous studies that reported that cattle farms located near a cattle abattoir had an increased risk of the disease [17,34]. Lindholm et al. [34] indicated that the shipment of FMD infected cattle to an abattoir during an outbreak could have contributed to the spread of the virus during transportation and at the site of the abattoir. With regard to the study area, cattle abattoirs are commonly located near main roads (see Figure 1). Since the main road was generally used by most dairy farmers for milk transportation and cattle feed shipping, contamination of the FMD virus from the ground to a farm vehicle could have occurred.

Previous reports indicated that limited vaccination coverage [29] or inconsistent FMD vaccination programs [35] were risk factors for FMD outbreaks. In this study, we examined the effects of vaccine administration because vaccination programs were applied three times a year to all dairy farms as recommended by the DLD [36]. However, the administration of the vaccine was done by different personnel. Our data revealed that a higher risk of FMD was found in dairy farms where vaccinations were administered by dairy cooperative staff as compared to DLD officers and livestock volunteers that were trained by DLD. It is possible that dairy cooperative staff might not have handled the vaccination procedure properly. Based on the results, we recommend that vaccinations should be administered by authorized individuals or trained persons. Furthermore, DLD officers should emphasize the quality of the vaccines and oversee the administration of the vaccines and the maintenance of the vaccine cold chain as has been described by Young et al. [37].

Farms with a history of an FMD outbreak in the previous 12 months had a lower risk of experiencing a new FMD outbreak (OR = 0.44). As has been discussed by Yano et al., [18] in northern Thailand, owners that were impacted by an FMD outbreak might be more aware of the prescribed preventative and control measures for an FMD outbreak. Such farmers were educated by DLD officers in order to improve biosecurity management practices, such as with the restriction of people entering the farm and the required use of disinfectants for cleaning vehicles [38]. Our data also supported the awareness of farmer experience with FMD outbreaks. We found that a high proportion of farmers who used disinfectants were farmers who had experienced an FMD outbreak in the previous 12 months (see Table S2). In addition, herd immunity due to a recent previous outbreak may decrease the risk of a subsequent outbreak, particularly from a similar strain of the virus as the outbreaks in 2016 and previous year were from the same serotype (Serotype O). A previous study also indicated that the risk of further outbreaks of the past outbreak herd was reduced due to individual immune [39].

In the present study, an effort was made to collect as much data as possible. We reviewed and compared FMD outbreak investigation data obtained from both district and provincial levels to ensure that we did not lose any FMD outbreak investigation data. The farm questionnaire interview was given to all dairy farmers. However, a limitation was found. Recall bias may have occurred because most of the farmers did not keep logbooks for farm-related activities, such as with regard to the recorded dates for vehicles entering the farm and critical information on any potential clinical signs along with the treatment of sick cows. The potential effects of recall bias may not high because the recall time was in the range of 2–11 months. In addition, regarding management we asked in the questionnaire, farmers provided the information that they normally perform the same management.

The goals of the national livestock strategic plan developed by the DLD included many prevention and control measures that were in accordance with the regional OIE campaign in Southeast Asia and China (SEACFMD). These measures were put in place to decrease FMD incidences, enhance biosecurity on farms and establish an FMD zone with a vaccination protocol [38,40]. The management risk factors reported in this study were consistent with the recommendations issued by the DLD for FMD prevention and control. The DLD should emphasize and educate dairy farmers in order to implement key farming activities that include farm husbandry, on-farm biosecurity, and immunization for FMD prevention and control at the farm level. Therefore, we recommend that dairy farmers should be intensively educated on issues of farm biosecurity. Vaccinations for FMD should be conducted by authorized or trained personnel. In addition, cooperation between farmers, dairy cooperative staff members and livestock officers should be strengthened for improved FMD surveillance.

## 5. Conclusions

This first analytical study identified the risk factors associated with the FMD outbreaks in dairy farms in Chiang Mai Province, northern Thailand. Farmers should follow the recommendations dictated by a comprehensive national livestock strategy by improving farm biosecurity management techniques. In addition, linking biosecurity and disease control practices with the engagement of stakeholders is recommended to support the effectiveness of a strategic FMD control plan.

## Figures and Tables

**Table 1:** Descriptive findings for general farm management practices, animal and vehicle movement, vaccination of animals and farm biosecurity in the study area.

Items	Percentage (Observation Number/Total Number)
Farm with tie-stall system	86.6% (387/447) ^a^
Use tap water on farm	91.1% (407/447) ^a^
Farm with waste management	77.2% (275/447) ^a^
Farms located within a 5 km radius of cattle abattoirs	22.8% (102/447) ^a^
Farms located within a 5 km radius of milk collecting center	70.5% (315/447) ^a^
Farms located near shared cattle grazing areas in a 10 km radius	25.7% (115/447) ^a^
Farm located near roadways	65.5% (293/447) ^a^
FMD infected carcass management	100% (18) ^b^
Entrance of vehicle carrying the stock and/or cow out of the farm	60.9% (272/447) ^a^
Entrance of vehicle carrying roughage feed for delivery	97.1% (434/447) ^a^
Entrance of dung trader vehicles	92.4%(413/447) ^a^
Employing artificial insemination (AI) services with more than 1 staff member	28% (126/447) ^a^
Vaccination for all cattle in the farm	93.5% (418/447) ^a^
Using disinfectant for vehicles and floor cleaning	86.1% (385/447) ^a^
Treatment of FMD infected cattle	97.1% (136/140) ^c^
Purchasing a new cow without quarantine	95.4% (84/88) ^d^

^a,b,c,d^ indicate denominators; ^a^ = total number of all farms, ^b^ = FMD outbreak farm which death animal, ^c^ = total number of FMD outbreak farms, ^d^ = total number of farms purchasing new stock.

**Table 2:** Variables with FMD outbreaks based on univariable logistic regression analysis (*p* < 0.2).

	Independent Variables	Level	Control	Case	*p*-Value	OR (95%CI)
1	Farm with tie-stall system	Tie-stall	271	116	0.11	1.55 (0.84, 2.81)
		Tie-stall and free area	36	24		
2	Use tap water on farm	No	32	8	0.1	1.91 (0.83, 4.95)
		Yes	275	132		
3	Farm with waste management	No	198	77	0.05	1.48 (0.96, 2.27)
		Yes	109	63		
4	Distance between farm and neighboring beef farm	>500 m	55	34	0.11	0.68 (0.40, 1.14)
		<500 m	252	106		
5	Farms located within a 5 km radius of cattle abattoirs	<5 km	250	95	0.001	2.07 (1.27, 3.35)
		>5 km	57	45		
6	Farms located near shared cattle grazing areas in a 10 km radius	No	237	95	0.036	1.6 (1.00, 2.55)
		Yes	70	45		
7	Farm located near roadways	No	117	37	0.015	1.71 (1.08, 2.74)
		Yes	190	103		
8	Purchasing of a new cow without following quarantine protocol	No	261	102	0.002	2.15 (1.29, 3.57)
		Yes	46	38		
9	Entrance of vehicle carrying the stock and/or cow out of the farm	No	129	46	0.065	1.47 (0.95, 2.31)
		Yes	178	94		
10	Entrance of dung trader vehicles	No	18	16	0.039	0.48 (0.22, 1.04)
		Yes	289	124		
11	FMD outbreak status in the previous 12 months	No	249	127	0.009	0.44 (0.21, 0.85)
		Yes	58	13		
12	Vaccination was done before outbreak within 4 months	No	133	49	0.09	1.41 (0.92, 2.20)
		Yes	174	91		
13	FMD vaccination administration by dairy cooperative staff members	No	261	111	0.13	1.48 (0.85, 2.54)
		Yes	46	29		
14	Farming area (District)	Mae On	206	74	0.002	^a^
		San Kamphaeng	67	35		
		San Sai	34	31		

^a^ Not available.

**Table 3:** Risk factors from the final logistic regression model for FMD outbreak in dairy farms.

	Risk Factors	Adjusted OR (95%CI)	*p*-Value
1	Purchasing of a new cow without following quarantine protocol	2.41 (1.45, 4.05)	<0.001
2	Farms located within a 5 km radius of cattle abattoirs	1.83 (0.99, 3.40)	0.055
3	FMD vaccination administration by dairy cooperative staff members	2.52 (1.39, 4.58)	0.003
4	Farms located near shared cattle grazing areas in a 10 km radius	1.83 (1.11, 3.02)	0.017
5	FMD outbreak status in the previous 12 months	0.44 (0.22, 0.86)	0.012
6	Farming area: Mae On	Reference	0.016
	San Kamphaeng	1.66 (0.87, 3.15)	
	San Sai	2.92 (1.41, 6.03)	

Hosmer–Lemeshow test (*p* = 0.08); Akaike information criteria (AIC) = 524.89.

## Supplementary Materials

The following data are available online at https://www.mdpi.com/2076-2615/10/3/512/s1, Table S1: Description of variables used in questionnaires, Table S2: Univariable analysis of risk factors and FMD outbreak in the study area, Table S3: Results from chi-square test show collinearity between a pair of risk factors.
