# Supplementary material for: Determination of Risk Factors Associated with Foot and Mouth Disease Outbreaks in Dairy Farms in Chiang Mai Province, Northern Thailand
Source: Animals (Basel). 2020 Mar 19;10(3):512. doi: 10.3390/ani10030512 (PMC7143784; doi:10.3390/ani10030512)
Supplement: Supplementary file 1 [file animals-10-00512-s001.zip › Table S3.docx]

**Table S3.** Results from chi-square test showing collinearity between a pair of risk factors.

| **Independent Variable 1** |  | **Independent Variable 2** | | ***p*-Value** |
| --- | --- | --- | --- | --- |
|  |  | Farm located near road ways | |  |
|  |  | No | Yes |  |
| Farms located within a 5 km radius of cattle abattoirs | No | 138 | 207 | 5.63x10^-6^ |
|  | Yes | 16 | 86 |  |
|  |  | Use tap water on farm | |  |
|  |  | No | Yes |  |
| Farms located near shared cattle grazing areas in a 10 km radius | No | 13 | 319 | 2.39x10^-10^ |
|  | Yes | 27 | 88 |  |
|  |  | Entrance of vehicle carrying the stock and/or cow out of the farm | |  |
|  |  | No | Yes |  |
| Purchasing of a new cow without following quarantine protocol | No | 158 | 201 | 2.17x10^-5^ |
|  | Yes | 17 | 71 |  |
