# Supplementary material for: Determination of Risk Factors Associated with Foot and Mouth Disease Outbreaks in Dairy Farms in Chiang Mai Province, Northern Thailand
Source: Animals (Basel). 2020 Mar 19;10(3):512. doi: 10.3390/ani10030512 (PMC7143784; doi:10.3390/ani10030512)
Supplement: Supplementary file 1 [file animals-10-00512-s001.zip › Table S1.docx]

**Table S1.** Description of variables used in questionnaires.

| **Variables** | **Descriptions** |
| --- | --- |
| **Farm management practices** | |
| Farm with tie-stall system | Type of tie-stall system in dairy farm  1 = tie stall, 2 = tie stall and free area |
| Use tap water on farm | Type of water source in dairy farm  1 = tap water, 2 = other sources (ground water, stream water, etc.) |
| Farm with waste management | Dairy farm managed waste production (i.e. cow dung, contaminated runoff) to prevent waste from accumulating in feeding, watering, and shade areas  1 = applicable, 2 = not applicable |
| **Farm location and environment** | |
| Distance between farm and neighboring dairy farm less than 500 m | This variable was defined as distance between farm and neighboring dairy farm  1 = equal or less than 500 m, 2 = greater than 500 m |
| Distance between farm and neighboring beef farm less than 500 m | This variable was defined as distance between farm and neighboring beef farm  1 = equal or less than 500 m, 2 = greater than 500 m |
| Farms located within a 5 km radius of cattle abattoirs | This variable was defined as distance between farm and cattle abattoirs  1 = equal or less than a radius of 5 km, 2 = greater than a radius of 5 km |
| Farms located within a 5 km radius of pig abattoirs | This variable was defined as distance between farm and pig abattoirs  1 = equal or less than a radius of 5 km, 2 = greater than a radius of 5 km |
| Farms located within a 5 km radius of milk collecting center | This variable was defined as distance between farm and milk collecting center  1 = equal or less than a radius of 5 km, 2 = greater than a radius of 5 km |
| Farms located near shared cattle grazing areas in a 10 km radius | This variable was defined as distance between farm and shared cattle grazing areas  1 = equal or less than a radius of 10 km, 2 = greater than a radius of 10 km |
| Farm located near road ways | This variable was defined as distance between farm and road ways  1 = equal or less than 500 m, 2 = greater than 500 m |
| **Animal and vehicle movement** | |
| Purchasing of a new cow and/or young stock without following quarantine protocol | Farmer purchase of a new cow or young stock without following quarantine protocol  1 = applicable, 2 = not applicable |
| Entrance of vehicle carrying the young stock and/or cow out of the farm | Vehicle enters to farm for carrying young stock and/or cow out of the farm  1= applicable, 2 = not applicable |
| Entrance of vehicle carrying roughage feed for delivery | Having vehicle movement for carrying roughage feed  1 = applicable, 2 = not applicable |
| Entrance of dung trader vehicles | Dung trader vehicle enters to farm  1 = applicable, 2 = not applicable |
| Type of raw milk transportation | Type of raw milk transportation from farm to milk collecting center  1 = own farm vehicle, 2 = publicly shared milk collecting vehicles |
| Using artificial insemination (AI) service from a specific staff | Using artificial insemination (AI) service from a specific staff  1 = specific staff, 2 = multiple staff |
| **Historical of FMD and vaccination status** | |
| FMD outbreak status in the previous 12 months | This variable was defined as dairy farm which historical of FMD in the previous 12 months prior to the outbreak window (July to November 2016)  1 = having previous FMD outbreak, 2 = no previous FMD outbreak |
| Vaccination was done within 4 months before FMD outbreak | Whether vaccination was done within 4 months before FMD outbreak  1 = applicable, 2 = not applicable |
| Vaccination for all cattle in the farm | Whether all cattle in the farm had been vaccinated  1 = yes, 2 = no (not all cattle in a farm were vaccinated) |
| FMD vaccination administration | Person who performed FMD vaccination in a study herd  1 = owner  2 = animal health volunteer  3 = veterinarian from Department of Livestock (DLD)  4 = dairy cooperative staff members |
| **Farm biosecurity during outbreak** | |
| Using disinfectant for vehicle and floor cleaning | Dairy farmer uses disinfectant for vehicle and floor cleaning during FMD outbreak  1 = applicable, 2 = not applicable |
| Treatment of FMD infected cattle | An FMD infected cattle was treated during outbreak  1 = applicable, 2 = not applicable |
| FMD infected carcass management | The disposal of dead animal carcasses due to FMD was performed under DLD protocol  1 = applicable, 2 = not applicable |
