# Supplementary material for: Determination of Risk Factors Associated with Foot and Mouth Disease Outbreaks in Dairy Farms in Chiang Mai Province, Northern Thailand
Source: Animals (Basel). 2020 Mar 19;10(3):512. doi: 10.3390/ani10030512 (PMC7143784; doi:10.3390/ani10030512)
Supplement: Supplementary file 1 [file animals-10-00512-s001.zip › Table S2.docx]

**Table S2.** Univariable analysis of risk factors and FMD outbreak in the study area.

| **Independent Variables** | **Level** | **FMD Non-Outbreak Farm** | **FMD Outbreak Farm** | ***p*-Value** | **OR (95%CI)** |
| --- | --- | --- | --- | --- | --- |
| **Farm management practices** | |  |  |  |  |
| Farm with tie-stall system | Tie stall | 271 | 116 | 0.11 | 1.55 (0.84, 2.81) |
|  | Tie stall and free area | 36 | 24 |  |  |
| Use tap water on farm | No | 32 | 8 | 0.1 | 1.91 (0.83, 4.95) |
|  | Yes | 275 | 132 |  |  |
| Farm with waste management | No | 198 | 77 | 0.06 | 1.48 (0.96, 2.27) |
|  | Yes | 109 | 63 |  |  |
| **Farm location and environment** | |  |  |  |  |
| Distance between farm and neighboring dairy farm less than 500 m. | No | 25 | 9 | 0.52 | 1.28 (0.56, 3.23) |
|  | Yes | 282 | 131 |  |  |
| Distance between farm and neighboring beef farm less than 500 m. | No | 55 | 34 | 0.11 | 0.68 (0.40, 1.14) |
|  | Yes | 252 | 106 |  |  |
| Farms located within a 5 km radius of cattle abattoirs | No | 250 | 95 | 0.0015 | 2.07 (1.27, 3.35) |
|  | Yes | 57 | 45 |  |  |
| Farms located within a 5 km radius of pig abattoirs | No | 139 | 57 | 0.36 | 1.2 (0.78, 1.84) |
|  | Yes | 168 | 83 |  |  |
| Farms located within a 5 km radius of milk collecting center. | No | 96 | 36 | 0.23 | 1.31 (0.82, 2.12) |
|  | Yes | 211 | 104 |  |  |
| Farms located near shared cattle grazing areas in a 10 km radius | No | 237 | 95 | 0.036 | 1.6 (1, 2.55) |
|  | Yes | 70 | 45 |  |  |
| Farm located near road ways | No | 117 | 37 | 0.015 | 1.71 (1.08, 2.74) |
|  | Yes | 190 | 103 |  |  |
| **Animal and vehicle movement** | |  |  |  |  |
| Purchasing of a new cow without following quarantine protocol | No | 261 | 102 | 0.002 | 2.15 (1.29, 3.57) |
|  | Yes | 46 | 38 |  |  |
| Entrance of vehicle carrying the stock and/or cow out of the farm | No | 129 | 46 | 0.065 | 1.47 (0.95, 2.31) |
|  | Yes | 178 | 94 |  |  |
| Entrance of vehicle carrying roughage feed for delivery | No | 10 | 3 | 0.72 | 1.53 (0.38, 8.82) |
|  | Yes | 297 | 137 |  |  |
| Entrance of dung trader vehicles | No | 18 | 16 | 0.039 | 0.48 (0.22, 1.04) |
|  | Yes | 289 | 124 |  |  |
| Type of raw milk transportation | Own vehicle | 241 | 104 | 0.32 | 1.26 (0.76, 2.06) |
|  | Shared vehicle | 66 | 36 |  |  |
| Having the artificial insemination (AI) service more than 1 staff | No | 216 | 106 | 0.24 | 0.76 (0.46, 1.22) |
|  | Yes | 91 | 34 |  |  |
| **Historical of FMD and vaccination status** | |  |  |  |  |
| FMD outbreak status in the previous 12 months | No | 249 | 127 | 0.009 | 0.44 (0.21, 0.85) |
|  | Yes | 58 | 13 |  |  |
| Vaccination was done before outbreak within 4 months | No | 133 | 49 | 0.09 | 1.41 (0.92, 2.20) |
|  | Yes | 174 | 91 |  |  |
| Vaccination for all cattle in the farm | No | 17 | 12 | 0.22 | 0.62 (0.27, 1.48) |
|  | Yes | 290 | 128 |  |  |
| FMD vaccination administration by owner | No | 105 | 54 | 0.37 | 0.82 (0.53, 1.28) |
|  | Yes | 202 | 86 |  |  |
| FMD vaccination administration by animal health volunteer | No | 275 | 128 | 0.54 | 0.8 (0.36, 1.67) |
|  | Yes | 32 | 12 |  |  |
| FMD vaccination administration by veterinarian from DLD | No | 260 | 117 | 0.76 | 1.08 (0.6, 1.92) |
|  | Yes | 47 | 23 |  |  |
| FMD vaccination administration by dairy cooperative staff members | No | 261 | 111 | 0.13 | 1.48 (0.85, 2.54) |
|  | Yes | 46 | 29 |  |  |
| **Farm biosecurity during outbreak** | |  |  |  |  |
| Using disinfectant for vehicle and floor cleaning | No | 61 | 1 | ^a^ | ^a^ |
|  | Yes | 246 | 139 |  |  |
| Treatment of FMD infected cattle | No | ^a^ | 4 | ^a^ | ^a^ |
|  | Yes | ^a^ | 136 |  |  |
| FMD infected carcass management | No | ^a^ | ^a^ |  |  |
|  | Yes | ^a^ | 18 |  |  |

^a^ Not available
